# A serous borderline ovarian tumour in a transgender male adolescent

**DOI:** 10.1038/s41416-020-01129-4

**Published:** 2020-10-27

**Authors:** Kate Millington, Katherine Hayes, Sarah Pilcher, Stephanie Roberts, Sara O. Vargas, Amanda French, Jennifer Veneris, Allison O’Neill

**Affiliations:** 1 Division of Endocrinology, Boston Children’s Hospital, Boston, MA USA; 2 Division of Gynecology, Boston Children’s Hospital, Boston, MA USA; 3 Department of Pathology, Boston Children’s Hospital, Boston, MA USA; 4 Division of Gynecologic Oncology, Dana-Farber Cancer Institute, Boston, MA USA; 5 Dana-Farber Cancer Institute, Boston Children’s Hospital, Boston, MA USA

**Keywords:** Paediatric research, Paediatrics

## Abstract

Here we present a transgender male adolescent with an androgen receptor-positive serous borderline ovarian tumour in the setting of testosterone treatment for medical gender transition. To our knowledge, this is the second report of borderline tumour in a transgender individual and the first in an adolescent, an age group in which borderline tumours are extremely rare. We discuss the specific considerations of treating ovarian tumours in the transgender male population, the incompletely understood role of androgens in the genesis of ovarian epithelial neoplasia, and an emphasis on assessing cancer risk in transgender patients based on patient anatomy.

## Background

Gender diversity is increasingly recognised and the number of patients presenting for gender-affirming care has increased. Transmasculine individuals, those who are designated female at birth and have a masculine gender identity, may elect treatment with exogenous testosterone to produce secondary sex characteristics in line with their gender identity.^1^ The effects of testosterone therapy on the ovaries is incompletely understood with conflicting data in the literature. Some studies report polycystic ovarian pathology following testosterone treatment, with others reporting no treatment sequelae.^2^ Independent of concerns surrounding testosterone treatment, many transmasculine individuals are electing to retain their uterus, fallopian tubes, and ovaries and require screening for malignancies involving these organs.

We present a trans-male adolescent with an androgen receptor (AR)-positive serous borderline tumour (SBT) discovered shortly after starting on testosterone therapy. Because of the small number of cases of ovarian neoplasms in adolescents, with even fewer in the transgender population, the potential role of sex steroids in the genesis and pathogenesis of these tumours is largely unknown. Gynaecologic and medical providers treating trans youth must take into account each patient’s goals with regard to hormone therapy and gender-affirming surgery while simultaneously assessing health risks associated with patient anatomy. This case report was prepared using the CARE guidelines.^3^

## Case presentation

A 17-year-old transgender male with obesity, anxiety, and no prior abdominal surgical history presented to the emergency room with acute right lower quadrant pain and nausea. Medications included fluoxetine, norethindrone acetate to achieve menstrual suppression, begun 7 months prior, and subcutaneous testosterone cypionate, begun 12 weeks prior. Past medical and gynaecologic history included normal development with menarche at age 13 years and regular menstrual cycles until initiation of testosterone and norethindrone acetate. Exam demonstrated a palpable, tender, mobile mass extending 4 cm above the umbilicus. Laboratory evaluation was reassuring. Ultrasound demonstrated a large right-sided mass with solid and cystic components and absent vascular flow.

Due to concern for adnexal torsion, urgent surgery was recommended. After counselling regarding the risk, benefits, and fertility implications of ovarian cystectomy versus salpingo-oophorectomy, the patient and his parents elected salpingo-oophorectomy. He expressed plans for future gender-affirming surgery, including gonad removal. Intra-operative examination under anaesthesia showed Tanner V pubic hair, hirsutism, and clitoromegaly. A midline vertical incision was made. The right adnexa was torted three times in the presence of a large right ovarian mass with an intact ovarian capsule. A right salpingo-oophorectomy was performed. Inspection of the uterus, left tube and ovary, omentum, and palpation of the upper abdomen, liver edge, diaphragm, and pelvic and para-aortic lymph nodes showed no gross abnormalities. An omental biopsy and pelvic washings were performed.

**Fig. 1:**
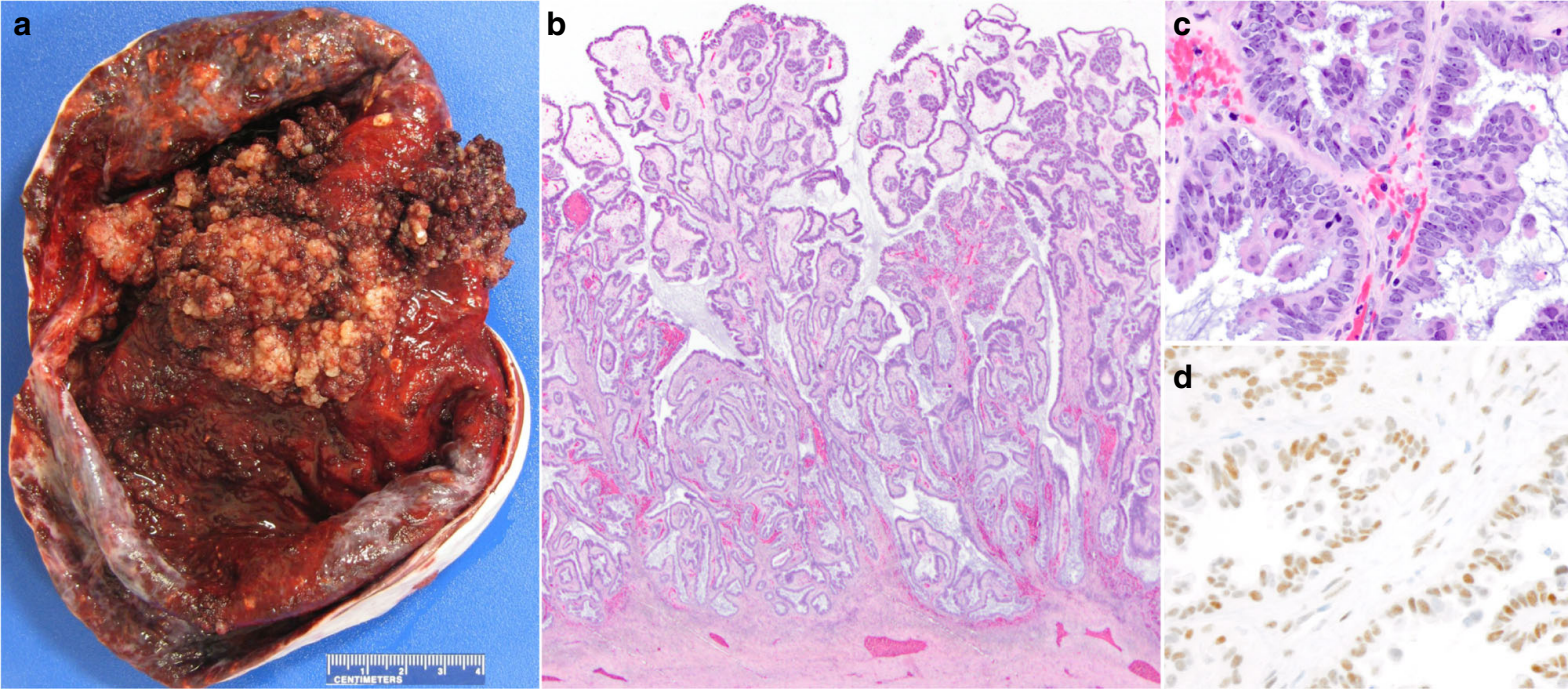
Pathologic features. Grossly, innumerable papillary projections lined the internal surface of the cyst (**a**). Microscopic papillary frond-like architecture and basophilic mucoid secretions (**b**; haematoxylin and eosin; original magnification, ×20) were composed of proliferating cuboidal to columnar epithelial cells with eosinophilic cytoplasm and absent nuclear atypia (**c**; haematoxylin and eosin; original magnification, ×600). Nuclear expression of androgen receptor was seen via immunohistochemistry (**d**; haematoxylin and eosin; original magnification, ×600).

Pathological examination showed a 15 × 14 × 9 cm tumour, with tan-red fleshy excrescences lining the internal surface (Fig. 1a). Histologically, there was a proliferation of cuboidal to columnar epithelial cells showing eosinophilic cytoplasm and arranged in papillary fronds (Fig. 1b, c). The tumour was confined to the ovary without evidence of invasion or implantation. Immunostaining for AR (AR441, DAKO, Carpinteria, CA) showed nuclear staining in lesional cells (Fig. 1d). A diagnosis of serous papillary borderline tumour (atypical proliferative serous tumour), International Federation of Gynecology and Obstetrics stage IA, was rendered.

Postoperative treatment, monitoring, and further gender-affirming hormone therapy were discussed by a multidisciplinary team involving paediatric endocrinology, oncology, gynaecology, pathology, and adult medical oncology. In the absence of peritoneal implants or extra-ovarian disease, the tumour was considered low risk. Given the potential for contralateral ovarian disease after removal of an SBT, surveillance of the remaining ovary was recommended. Testosterone was restarted 2 months following the surgery after discussing risks and benefits with the patient and his family. He continued on norethindrone acetate daily to obtain adequate menstrual suppression. Surveillance ultrasound of the remaining ovary obtained 6 months postoperatively was normal.

## Discussion

We present an SBT in a trans-male adolescent receiving testosterone for medical gender transition. The sole previous report of SBT in a transmasculine individual occurred in a 38-year-old receiving testosterone therapy.^4^ Other ovarian neoplasms reported in this setting include one serous cystadenoma,^4^ two mature cystic teratomas,^5^ and one endometrioid adenocarcinoma.^6^

Ovarian epithelial tumours are rare in adolescents and carry a more favourable 10-year survival rate than the same diagnoses in adults.^7^ SBTs are non-invasive ovarian epithelial tumours distinct from frank carcinoma. In our paediatric hospital, borderline tumours account for <1% of ovarian neoplasms among patients aged <21 years.^8,9^ The overall survival for patients with stage 1 disease does not differ from the general population.

The role of testosterone treatment in tumour progression is uncertain as a putative role for androgens and the AR in the development or proliferation of ovarian tumours has not been established. Androgen signalling has a role in granulosa cell maturation and differentiation, although in vitro exposure of ovarian cancer cell lines to androgens does not result in cell proliferation.^10^ Patients with polycystic ovary syndrome (PCOS), who are exposed to high levels of endogenous androgens, have not shown an increased risk in ovarian cancer overall; however, Olsen et. al. showed a modest increased risk of SBT in overweight women with PCOS and high circulating androgens (odds ratio 2.6, 95% confidence interval 1.0–6.1).^11^ Clinical studies have failed to show an association between elevated androgen levels and ovarian cancer risk,^12^ and attempts to treat chemotherapy-refractive ovarian tumours with androgen deprivation have yielded moderate responses at best in small numbers of patients.^13^ Prior studies investigating endogenous androgens may not be applicable to exogenous testosterone treatment given to achieve male range levels and, in the case of transgender youth, for pubertal induction.

The presence of a sex hormone-sensitive cancer is a contraindication to testosterone therapy, but there are no formal recommendations for the use of testosterone in patients with SBT. Given the importance of gender-affirming therapy, which has been shown to reduce suicide risk and improve overall well-being,^14^ our multi-disciplinary team carefully weighed the risks and benefits of restarting testosterone therapy and, in the context of a completely resected tumour and ambiguous risks associated with endogenous steroids, ultimately recommended restart. Appropriate tumour surveillance was also unclear as there is no data to guide management in this area. The team recommended at least 5 years of periodic transvaginal ultrasounds of the contralateral ovary unless oophorectomy was completed sooner. While transvaginal ultrasounds were deemed of higher sensitivity, transabdominal were prioritised given patient preference.

Attempts to estimate the prevalence of reproductive cancers in the transgender population have been unsuccessful. Current guidelines suggest routine screening based on retained internal organs in line with cisgender screening recommendations.^15^ Keeping in mind that significant barriers to care exist for transgendered persons, all providers must be cognizant of and consider the health risks posed by each patient’s anatomy, for example, that transmasculine patients may retain their ovaries, and should screen patients appropriately.

This manuscript details the first reported borderline ovarian tumour in a transmasculine adolescent receiving testosterone. The role that testosterone treatment played in the development, growth, and recurrence risk of his tumour is largely unknown. Further study regarding the prevalence and management of ovarian tumours in transmasculine individuals is needed. We propose the creation of a tumour registry for reproductive tract tumours in the transgender population to gather longitudinal data and increase our understanding of the role that gender-affirming hormones play in the origin and progression of these tumours.

## Data Availability

Clinical data from this case was abstracted from the electronic medical record in a de-identified manner.
